# 3D Tensor Based Nonlocal Low Rank Approximation in Dynamic PET Reconstruction

**DOI:** 10.3390/s19235299

**Published:** 2019-12-01

**Authors:** Nuobei Xie, Yunmei Chen, Huafeng Liu

**Affiliations:** 1 State Key Laboratory of Modern Optical Instrumentation, College of Optical Science and Engineering, Zhejiang University, Hangzhou 310027, China; xnb@zju.edu.cn; 2 Department of Mathematics, University of Florida Gainesville, Gainesville, FL 118105, USA; yun@math.ufl.edu

**Keywords:** dynamic positron emission tomography (PET), non-local, tensor decomposition, low-rank approximation, compressed sensing, reconstruction, distributed optimization

## Abstract

Reconstructing images from multi-view projections is a crucial task both in the computer vision community and in the medical imaging community, and dynamic positron emission tomography (PET) is no exception. Unfortunately, image quality is inevitably degraded by the limitations of photon emissions and the trade-off between temporal and spatial resolution. In this paper, we develop a novel tensor based nonlocal low-rank framework for dynamic PET reconstruction. Spatial structures are effectively enhanced not only by nonlocal and sparse features, but momentarily by tensor-formed low-rank approximations in the temporal realm. Moreover, the total variation is well regularized as a complementation for denoising. These regularizations are efficiently combined into a Poisson PET model and jointly solved by distributed optimization. The experiments demonstrated in this paper validate the excellent performance of the proposed method in dynamic PET.

## 1. Introduction

Positron emission tomography (PET) seeks to obtain radioactivity distributions by collecting numerous photons emitted by the annihilations of positrons that come from the isotope-labeled tracer injected in living tissue. Correspondently, considering the unique traits of physiological and molecular imaging, PET is universally and irreplaceably required in clinic imaging, especially in cancer diagnoses [1], lesion detection [2], and functional supervision in vivo. However, despite its predominance for functional imaging, PET is dwarfed in resolution due to limitations either from acquisition time or the injection dose when compared with other structural medical imaging systems such as magnetic resonance imaging (MRI) and computed tomography (CT). Moreover, in dynamic PET imaging, where the time-varying radioactivity concentration at each spatial location is obtained, the structural information and denoising performance are more critical, along with increasing demands for time activity curves (TAC) for different regions of interest (ROI). Under this framework, reliable algorithms for dynamic PET reconstruction have been discussed and debated for decades.

Initial attempts at PET reconstruction included the famous analytic filtered back-projected (FBP) [3] based algorithms, least squares (LS) [4], and the maximum likelihood-expectation maximization (ML-EM) [5] method. Although milestones, problematic conditions [6] always accompany the optimization of the mentioned algorithms (i.e., the solution might be sensitive to trivial fluctuation and thus consequently increase noise along the iterations). Thus, much effort has been devoted to this topic, among which the maximum a posteriori (MAP) [7] strategy, or penalized ML method [6], have become the key to the solution. The main idea for this strategy is to integrate regularization terms, or image priors from a Bayesian perspective, into the reconstruction model. There are various representative regularizations, including the weighted quadratic penalty [8], Gibbs prior [9], Gauss–Markov prior [10], Huber prior [11], total variation [12], and so on. More recently, especially for dynamic imaging, temporal priors are starting to be introduced in reconstruction (e.g., the tracer kinetics model [13,14,15], kernel model [16], and dose estimation [17]).

Nevertheless, in spite of the significant progress achieved by researchers, traditional reconstruction algorithms are still open to question. For one thing, traditional methods, to a large extent, only focus on denoising behavior while ignoring the structural information within the images. This has undoubtedly created a greater divide within the increasing clinical demand for resolution. The other problem, which is of greater significance, is that the trade-off between the temporal and the spatial resolution constantly degrades the quality of the dynamic reconstruction (i.e., a better temporal resolution requires more frequent sampling within a given duration, which causes fewer photon counts for each frame, thus undermining the spatial resolution, and vice versa).

**Figure 1:**
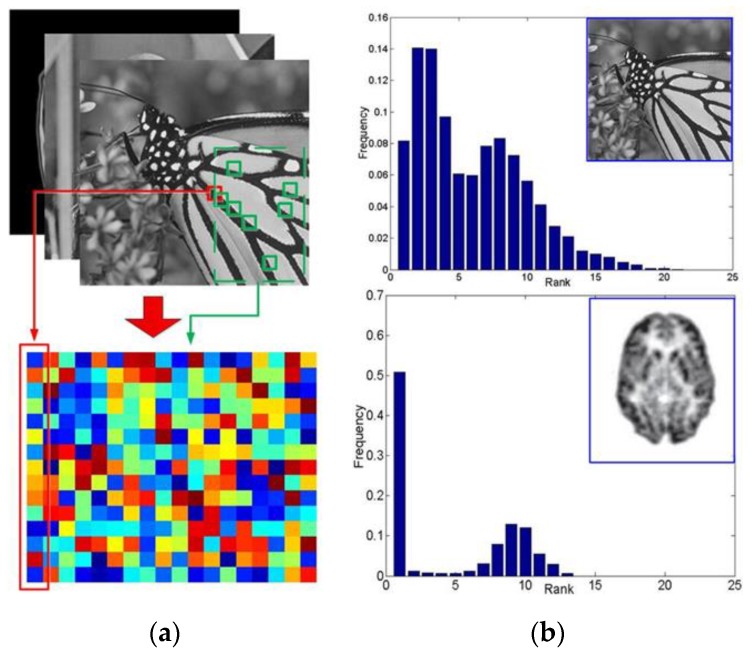
The nonlocal low-rank validation on images. For each random-selected exemplar patch (e.g., 5 × 5 sized), there can be found ample similar patches within the image itself. (**a**) By stretching the exemplar patch and its similar patches into vectors, a corresponding feature matrix (e.g., 25 × 30 sized) can be formulated. (**b**) The rank distribution for the matrices which is formulated from given images. Up: the monarch; Down: Positron emission tomography (PET) scan of an Alzheimer’s patient’s brain.

In this paper, we explore a potential solution for the mentioned dilemma by providing a novel tensor based nonlocal low-rank framework in dynamic PET reconstruction. Several efforts have been devoted to related topics. Low rank decomposition [18] and non-negative matrix factorization [19,20,21], on the one hand, have been found to be able to capture the inner temporal correlation in a dynamic PET. On the other hand, the nonlocal feature, which refers to the abundant self-similar structures within an image, are excellently adopted in image denoising (e.g., the nonlocal means (NLM) [22], weighted nuclear norm minimization (WNNM) [23], block-matching 3D denoising (BM3D) [24], nonlocal restoration [25], and nonlocal representation [26]). Moreover, in [27], Dong et al. demonstrate the existence of nonlocal self-similarities and sparse features in MRI images. As shown in Figure 1, nonlocal low-rank [28,29] features exist not only in ordinary natural images but also in PET reconstructed images. Furthermore, as will be illustrated in Section 3, we promote the use of matrix based nonlocal low-rank features in a novel tensor form and successfully apply it to dynamic PET reconstruction. In this way, the structures are not only enhanced spatially by the image itself but simultaneously completed by relevant frames across the temporal dimension.

The main contributions are listed as follows:

(1) An innovative form of a nonlocal low rank tensor constraint is adopted in the Poisson’s model, which captures data correlation in multiple dimensions in dynamic PET, beyond just spatiotemporal correlation. For one thing, without any additional information, Poisson’s model exploits the temporal information among the frames themselves, effectively complementing the structures in low-active frames and recovering severely corrupted data. For the other, it exploits the spatial information from nonlocal self-similarities within each frame, thereby enhancing the structured sparsity for each image.
(2) As an additional regularization, the total variation (TV) constraint is employed to extract local structure and further complement the denoising function. On the other hand, the expectation–maximization method is employed as a fidelity term to incorporate hidden data in the objective function and thus increase efficiency in optimization.
(3) In the optimization procedure, we develop a distributed optimization framework inspired by the alternative direction method of multipliers (ADMM) [30]. In this way, the mentioned terms can be explicitly organized in a united objective function and effectively handled as three subproblems during the iterations.

## 2. Background

### 2.1. Dynamic PET Imaging Model

Typically, during the detection procedure, the PET scanner collects the emitted photons and then pre-processes them into so-called sinogram data as the input of the reconstruction. For dynamic PET, let the sinogram matrix $Y=\left[y_{1},y_{2},\ldots ,y_{t},\ldots ,y_{T}\right]\in {\mathbb{R}}^{M\;\times \;T}$ denote the collection of sinogram data vectors, where t=1,2,…,T denotes the index of the frames; and sinogram vector $y_{t}$ = $\{y_{tq\;}$, $q=1,\ldots ,M\}\in {\mathbb{R}}^{M}$ denotes the sum of the photons collected in the *t*-th frame, where *q* represents the index of the total *M* pairs of detectors. Correspondently, $X=\left[x_{1},x_{2},\ldots ,x_{t},\ldots x_{T}\right]$ $\in {\mathbb{R}}^{N\;\times \;T}$ denotes the collection of the images that are supposed to be recovered, where vector $x_{t}\in {\mathbb{R}}^{N}$ represents the *t*-th frame. Since Poisson distribution uses inherited PET systems, the reconstruction of each frame can be successfully modeled by the affine transformation:

(1) $$y_{t}∼\mathrm{Poisson}\left\{{\bar{y}}_{t}\right\}\;s.t.\;{\bar{y}}_{t}=E\left(y_{t}\right)=Gx_{t}+r_{t}+s_{t},$$

where ${\bar{y}}_{t}$ is the expectation of $y_{t}\;$; $G\in {\mathbb{R}}^{M\times N}$ is the system matrix; and $r_{t}$ and $s_{t}$ represent the *random coincidence* and *scatter coincidence*, which inevitably contain heavy noise. In this way, we can obtain the likelihood function of $y_{t}$ as

(2) $$\mathrm{Pr}(y_{t}|x_{t})=\prod_{q}^{M}e^{-{\bar{y}}_{tq}}\frac{{\bar{y}}_{tq}{}^{y_{tq}}}{y_{tq}!}.$$

Instead of maximizing Label (2), we estimate $x_{t}$ by minimizing the negative log-likelihood version for the convenience of optimization:

(3) $$\underset{x_{t}}{\min}P\left(x_{t}\right)=\underset{x_{t}}{\min}-\log(\mathrm{Pr}(y_{t}|x_{t}))=\underset{x_{t}}{\min}\sum_{q}^{M}{\bar{y}}_{tq}-y_{tq}\log({\bar{y}}_{tq})\;s.t.\;{\bar{y}}_{t}=Gx_{t}+r_{t}+s_{t},$$

where the constant term $\log(y_{tq}!)$ is left out.

Therefore, in the scale of dynamic reconstruction, Equation (3) can be transformed into

(4) $$\underset{X}{\min}P\left(X\right)=\underset{X}{\min}\sum_{t}^{T}\sum_{q}^{M}{\bar{y}}_{tq}-y_{tq}\log({\bar{y}}_{tq})\;s.t.\;{\bar{y}}_{t}=Gx_{t}+r_{t}+s_{t}.$$

However, optimization merely by Equation (4) is ill-conditioning—i.e., the reconstruction is vulnerable to accumulated iterative noise accompanying the iteration. The predominant solution to this shortcoming is to include the regularization terms in Equation (4) as the image prior. Thus, the object function of PET reconstruction can be written as

(5) $$\underset{X}{\min}\Psi \left(X\right)=\underset{X}{\min}P\left(X\right)+R\left(X\right),$$

where P(X) denotes the fidelity term defined in Equation (4), and R(X) denotes the regularization term.

### 2.2. Tensor Decomposition

Just like the matrix, the low-rank approximation of the tensor is also inevitably based on tensor decomposition. At present, the strategies for tensor decomposition mainly fall into three groups: the CANDECOMP/PARAFAC [31] (CP) decomposition methods, Tucker decomposition [32,33] methods, and tensor-singular value decomposition [34,35,36] (t-SVD) methods. Because of the stability and efficiency inherited in their optimization, t-SVD has aroused increasing interest among the community.

**Figure 2:**
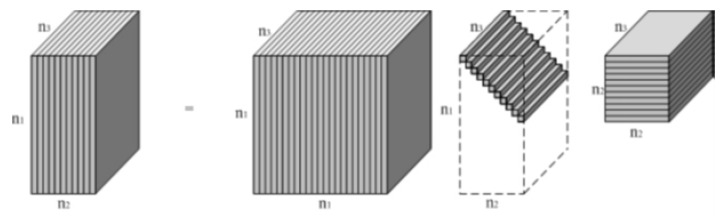
Illustration of tensor-singular value decomposition (t-SVD) upon an $n_{1}\times n_{2}\times n_{3}$ tensor [35].

As shown in Figure 2, for a three-way tensor $𝓐\in {\mathbb{R}}^{n_{1}\times n_{2}\times n_{3}}$, t-SVD shares the same form as the matrix SVD:

(6) $$𝓐=𝓤*𝓢*{𝓥}^{T},$$

where $𝓤\in {\mathbb{R}}^{n_{1}\times n_{1}\times n_{3}}$ and $𝓥\in {\mathbb{R}}^{n_{2}\times n_{2}\times n_{3}}$ are *orthogonal tensors* [37]; ${𝓥}^{T}$ represents the *conjugate transpose* [34] of 𝓥; $𝓢\in {\mathbb{R}}^{n_{1}\times n_{2}\times n_{3}}$ is a *f-diagonal tensor* in which each frontal slice ${𝓢}^{\left(i\right)}$ is a diagonal matrix [35]; and * denotes the *t-product* [37,38].

In this paper, we proposed a nonlocal tensor low-rank framework for dynamic PET reconstruction, where the tensor low-rank approximation is efficiently and effectively conducted by a t-SVD based method. The details of this method will be illustrated in Section 3.

## 3. Method

In this paper, we proposed a novel reconstruction framework that can jointly recover, denoise, and (mostly) critically complete the structures in the dynamic PET imaging system. The overall procedure is illustrated in Algorithm 1.


**Algorithm 1: Dynamic PET reconstruction via Nonlocal Low-rank Tensor Approximation and Total Variation**
**Input:** Sinogram Y and system matrix G, weighting parameters α,β,λ,η, and the reference frame index $t_{r}$. 1: **Initialization:**
$k=0,X^{0}=\mathrm{FBP}\left(Y\right)$.2: **Repeat:**3:  Compute the patch sets $S_{tri}{}^{\left(k\right)},\;\forall i$ based on the $t_{r}$-th frame $x_{tr}{}^{\left(k\right)}$ using (7).                         ⊳𝓛−subproblem
4:  Construct the nonlocal featured tensor ${𝓧}_{i}{}^{\left(k+1\right)},\forall i$ by (8) and (9). 5:  Approximate the low-rank tensor ${𝓛}_{i}{}^{\left(k+1\right)},\forall i$ by adopting t-SVT method in (13) and (21).6:  Construct Ω by updating the differential vector $\omega_{t}{}^{\left(k+1\right)},\forall t$ via (22).                        ⊳ω−subproblem 7:  Update the Lagrangian multiplier $v_{t}{}^{\left(k+1\right)},\forall t$ via (23).8:  **Repeat**:                   ⊳X−subproblem
9:    E-step: Introduce the expectation variable ${\hat{c}}_{tqj}$ and construct the X−relevant objective function Ψ(X) in (24).10:    M-step: Update $x_{tj}^{\left(k+1\right)},\forall t,j$ using (25) and (26).11:  **Until**: Inner Relative change $\frac{\left(X^{\left(k+1\right)}-X^{\left(k\right)}\right)}{X^{\left(k+1\right)}}<10^{-5}$
12:k←k+1
13: **Until**: Relative change $\frac{\left(X^{\left(k+1\right)}-X^{\left(k\right)}\right)}{X^{\left(k+1\right)}}<10^{-6}$
14: **Output:** Reconstructed image sequence $X^{\left(k\right)}$.

### 3.1. Nonlocal Low Rank Tensor Approximation

The nonlocal tensor regularization consists of two parts: forming the nonlocal tensor within the recovered frames and formulating the low-rank property in the formed tensors.

#### 3.1.1. Tensor Formulation

During the optimizing procedure, a temporary estimated image sequence $X=\left[x_{1},\;x_{2},\;\ldots ,x_{t},\ldots x_{T}\right]\in {\mathbb{R}}^{N\times T}$ will be obtained after each iteration, where $x_{t}\in {\mathbb{R}}^{N}$ represents the *t*-th frame of image vector as mentioned in Section 2.1. Similar to Figure 1, numerous *W* × *W* sized overlapping patches ${\tilde{x}}_{ti}\in {\mathbb{R}}^{n}$ (*n* = *W* × *W*) can be extracted from each $x_{t}$, where *i* denotes the index of the image patch. According to the nonlocal self-similar properties, within the image, there are plentiful patches that share the same structure with each ${\tilde{x}}_{ti}$. Based on the *Euclidean* distance, we choose the *m* nearest patches for each ${\tilde{x}}_{ti}$:

(7) $$S_{ti}=\{s|\|{\tilde{x}}_{ti}-{\tilde{x}}_{ti,s}{\|}_{2}<\rho_{ti}\},$$

where $S_{ti}$ is the index set of similar patches for the *i*-th positioned patch ${\tilde{x}}_{ti}$; and $\rho_{ti}$ is the threshold value defined by the distance between ${\tilde{x}}_{ti}$ and its *m*-th nearest patch. Thus, for each exemplar ${\tilde{x}}_{ti}$, we formulate a matrix:

(8) $$X_{ti}=\left[{\tilde{x}}_{ti},{\tilde{x}}_{ti,1},\ldots ,{\tilde{x}}_{ti,s},\ldots ,{\tilde{x}}_{ti,m-1}\right],X_{ti}\in {\mathbb{R}}^{n\;\times \;m}.$$

As shown in Figure 1, due to the nonlocal self-similarity, it is fairly assumed that each $X_{ti}$ is low-rank. 

From a dynamic recovery perspective, sets $S_{ti}$ for t=1,2,…,T are identical, since the structure in dynamic PET is unchanged. Therefore, based on the patch position *i*, we can construct a 3D tensor ${𝓧}_{i}\in {\mathbb{R}}^{n\;\times \;m\;\times \;T}$, whose frontal slices are calculated as

(9) $${𝓧}_{i}\left(:,:,t\right)=X_{ti},\;t=1,2,\ldots ,T.$$

**Figure 3:**
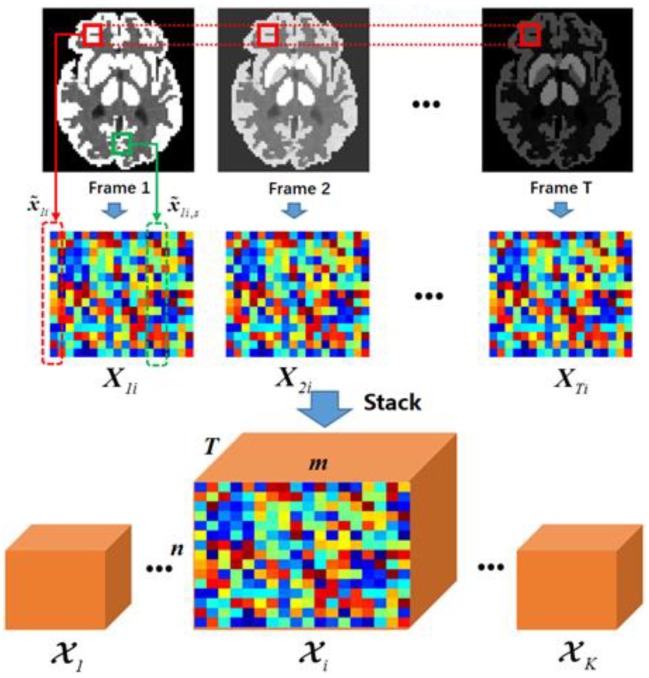
Illustration of tensor construction. The first row represents the temporary recovered image sequence. For the randomly chosen patch position *i*, similar patches can be found within its own frame and then grouped into matrices in the second row. By stacking the matrices along the frames, we managed to construct a feature tensor ${𝓧}_{i}\in {\mathbb{R}}^{n\times m\times T}$ for position *i*.

Figure 3 illustrates the overall procedure for forming a nonlocal tensor. Within each iteration, numerous ${𝓧}_{i}$s for various *i* positions will be constructed and then approximated by a low-rank property.

#### 3.1.2. Low Rank Tensor Approximation

The next step is the low rank approximation for constructed tensors ${𝓧}_{i},\forall i$. Traditionally, the primal model of rank regularization searches for the tensor ${𝓛}_{i}$, where

(10) $${𝓛}_{i}=\underset{{𝓛}_{i}}{\arg}minrank\left({𝓛}_{i}\right)\;s.t.\;{𝓛}_{i}={𝓧}_{i}.$$

Nevertheless, this model is non-deterministic polynomial-time (NP) hard, and its direct optimization is nonconvex. Taking this situation into account, we choose a surrogate form of Equation (10) and turn it into a convex optimization issue:

(11) $${𝓛}_{i}=\arg\underset{{𝓛}_{i}}{\min}\frac{1}{2}\|{𝓛}_{i}-{𝓧}_{i}{\|}_{F}^{2}+\lambda \|{𝓛}_{i}{\|}_{*}.$$

Here, ${\left\|\cdot \right\|}_{*}$ denotes the tensor nuclear norm [39] and ${\left\|\cdot \right\|}_{F}$ denotes the Frobenius norm. Under this circumstance, it is feasible to get a closed-form solution by adopting tensor singular value thresholding (t-SVT) [39]:

(12) $${𝓛}_{i}={𝓤}_{i}*D_{\lambda }\left({𝓢}_{i}\right)*{𝓥}_{i}{}^{T},$$

with ${𝓧}_{i}={𝓤}_{i}*{𝓢}_{i}*{𝓥}_{i}{}^{T}$ representing the mentioned t-SVD of ${𝓧}_{i}$ and

(13) $$D_{\lambda }\left({𝓢}_{i}\right)\;=\;IFFT_{\left(3\right)}\left({\left({\bar{𝓢}}_{i}-\lambda \right)}_{+}\right),$$

where $\left(X{)}_{+}=\max(X,0\right)$; $IFFT_{\left(3\right)}\left(X\right)$ denotes the fast Inverse Fast Fourier Transform (IFFT) of *X* across dimension 3; and ${\bar{𝓢}}_{i}=FFT_{\left(3\right)}\left({𝓢}_{i}\right)$ denotes the Fast Fourier Transform (FFT) of ${𝓢}_{i}$ across dimension 3. 

### 3.2. Total Variation Regularization in Dynamic PET

Apart from nonlocal low-rank tensor regularization, we also incorporate the total variation [40] (TV) as a complementary constraint into the dynamic PET reconstruction framework. Unlike the nonlocal tensor, the adopted TV regularization focuses on inherited local information within each frame. Moreover, this pixel-based regularization compromises patch-based regularization and thus improves the denoising performance of the proposed algorithm.

For each image frame $x_{t}\in {\mathbb{R}}^{N}$ in the estimated sequence $X=\left[x_{1},x_{2},\ldots ,x_{t},\ldots x_{T}\right]\in {\mathbb{R}}^{N\;\times \;T}$, we adopt an *l*_2_ formed TV regularization, which is properly formulated in accordance with augmented Lagrangian optimization [41,42]:

(14) $$\underset{x_{tj}}{min}\sum_{j}\|\omega_{tj}{\|}_{2}\;s.t.\;D_{j}x_{t}=\omega_{tj}\;\mathrm{for}\;\mathrm{all}\;j,$$

where $\omega_{tj}=D_{j}x_{t}$ denotes the discrete gradient of $x_{t}$ at position *j*; and $D_{j}$ denotes the *j*-th element of the corresponding differential operator D. Correspondingly, the augmented Lagrangian function can be written as:

(15) $$\underset{\Omega }{\min}\mathrm{DTV}\left(\Omega |X\right)\;=\sum_{t}^{T}\|\omega_{t}\|+\frac{\eta }{2}\|\omega_{t}-Dx_{t}{\|}_{2}^{2}-\nu_{t}\left(\omega_{t}-Dx_{t}\right),$$

where $\Omega =\left[\omega_{1},\omega_{2},\ldots ,\omega_{T}\right]\in {\mathbb{R}}^{N\;\times \;T}$ represents the collection of discrete gradient vectors $\omega_{t}=Dx_{t}$ for each recovered frame; η represents the tunable weighting parameters of the quadratic term; and $\nu_{t}$ represents the updatable multiplier vector. Consequently, this method guarantees the convexity of TV regularization, and hence equips the proposed method with global convergence.

### 3.3. Expectation Maximization for Fidelity Term

Indisputably, solving the fidelity term Equation (4), to a large extent, is an essential mission in estimating the reconstruction images X. Regardless of regularization, Equation (4) can be further written as:

(16) $$\underset{X}{\min}P\left(X\right)=\underset{X}{\min}\sum_{t=1}^{T}\sum_{j}^{N}\sum_{q}^{M}\left(g_{qj}x_{tj}-c_{tqj}\log(g_{qj}x_{tj}\right))\;\;s.t.\;y_{tq}=\sum_{q}c_{tqj},$$

where $x_{tj}$ denotes the *j*-th pixel in image $x_{t}$; $g_{qj}$ is the *qj*-th entry in system matrix ***G***, representing the contribution of the *j*-th pixel given to the *q*-th detector; and the hidden variable $c_{tqj}$ represents the photon count from the j-th pixel to the q-th detector pair in the t-th frame. 

The main challenge lies in the solving procedure, especially handling the hidden variable $c_{tqj}$. In this work, we adopt the well-known expectation maximization (EM) [5,43], which introduces ‘complete data’ into the model and thus facilitates optimization. In order to solve Equation (18), there are two essential steps:

- **E-step:** This step employs the expectation ${\hat{c}}_{tqj}$ = $E\left(c_{tqj}|x_{t},y_{tq}\right)$ as the substitute for the hidden variable $c_{tqj}$ in Equation (18):
  (17) $${\hat{c}}_{tqj}=\frac{g_{qj}x_{tj}}{\sum_{j}^{N}g_{qj}x_{tj}+r_{tq}+s_{tq}}y_{tq\;.}$$
- **M-step:** This step maximizes the likelihood by zeroing the derivative of Equation (19):
  (18) $$\frac{\partial P\left(X\right)}{\partial x_{tj}}=0.$$

The EM algorithm, which will be illustrated in greater detail in the next section, makes our proposed framework readily and efficiently solvable.

### 3.4. The Overall Optimization Framework

Based on Equation (5), the overall reconstruction model can be represented as

(19) $$\underset{X}{\min}\Psi \left(X\right)=\underset{X}{\min}\alpha \mathrm{TNL}\left(X\right)+\beta \mathrm{DTV}\left(X\right)+P\left(X\right),$$

where P(X) is the fidelity term in the reconstruction model Equation (16); TNL(X) represents the tensor formed nonlocal low rank constraint in Section 3.1; DTV(X) represents the dynamic PET adopted total variation term in Section 3.2; and α and β denote the weighting parameters. By taking the mentioned terms into account, the objective function can be formulated as:

(20) $$\Psi \left(X,𝓛,\omega \right)=\alpha \left(\sum_{i}\frac{1}{2}\|{𝓛}_{i}-{𝓧}_{i}{\|}_{F}^{2}+\lambda \|{𝓛}_{i}{\|}_{*}\right)+\beta (\sum_{t}^{T}\|\omega_{t}\|\;+\frac{\eta }{2}\|\omega_{t}-Dx_{t}{\|}_{2}^{2}-\nu_{t}\left(\omega_{t}-Dx_{t}\right))+\sum_{t}^{T}\sum_{q}^{M}{\bar{y}}_{tq}-y_{tq}\log({\bar{y}}_{tq}),\;\;s.t.\;{\bar{y}}_{t}=Gx_{t}+r_{t}+s_{t}.$$

Normally, recovering $X=\left[x_{1},x_{2},\ldots ,x_{t},\ldots x_{T}\right]\in {\mathbb{R}}^{N\times T}$ directly from Equation (20) is a complex process. For this process, we refer to the alternative direction method of multipliers (ADMM) [30] and divide the model into three subproblems of 𝓛, ω and X in a distributed optimization way.

**(1)**  𝓛**-subproblem.** Let ${\left(\cdot \right)}^{\left(k\right)}$ denote the updated variable after the *k*-th iteration; e.g., $X^{\left(k\right)}$ represents the computed image sequence after the *k*-th iteration. In the (*k* + 1)-th iteration, nonlocal low-rank approximation is first implemented. After the formulation of nonlocal feature tensors $X_{i}{}^{\left(k+1\right)},\forall i$, as illustrated in Section 3.1, we can obtain the function related to each low rank tensor:
  (21) $${𝓛}_{i}{}^{\left(k+1\right)}={𝓤}_{i}{}^{\left(k+1\right)}*D_{\lambda }\left({𝓢}_{i}{}^{\left(k+1\right)}\right)*{𝓥}_{i}^{\left(k+1\right)}{}^{T},$$
  with ${𝓧}_{i}{}^{\left(k+1\right)}={𝓤}_{i}{}^{\left(k+1\right)}*{𝓢}_{i}{}^{\left(k+1\right)}*{𝓥}_{i}^{\left(k+1\right)}{}^{T}$
**(2)**  ω**-subproblem.** Unlike updating ${𝓛}_{i}$, the discrete gradient is updated frame by frame. As shown in Equation (15), we update $\Omega =\left[\omega_{1},\omega_{2},\ldots ,\omega_{T}\right]\in {\mathbb{R}}^{N\times T}$ by employing the shrinkage operator [44]:
  (22) $$\omega_{t}{}^{\left(k+1\right)}=\max\left\{\|Dx_{t}{}^{\left(k\right)}-\frac{\nu_{t}{}^{\left(k\right)}}{\eta }{\|}_{2}-\frac{1}{\eta },0\right\}\frac{Dx_{t}{}^{\left(k\right)}-\nu_{t}{}^{\left(k\right)}/\eta }{\|Dx_{t}{}^{\left(k\right)}-\nu_{t}{}^{\left(k\right)}/\eta {\|}_{2}}\;.$$
  Correspondingly, the multiplier vector updates by:
  (23) $$v_{t}{}^{\left(k+1\right)}=v_{t}{}^{\left(k\right)}-\eta \left(Dx_{t}{}^{\left(k\right)}-\omega_{t}{}^{\left(k+1\right)}\right).$$
**(3)**  X**-subproblem.** After the update of 𝓛 and ω, the last critical process is to update $X=\left[x_{1},x_{2},\ldots ,x_{t},\ldots x_{T}\right]\in {\mathbb{R}}^{N\times T}$. In addition to the fidelity term in Equation (18), the former mentioned regularizations must be considered. In this procedure, we adopt the EM algorithm and hence reform Equation (20) into a joint function relevant to X (or $x_{tj}$):
  (24) $$\Psi \left(X\right)=\sum_{t}^{T}\sum_{j}^{N}\sum_{q}^{M}\left(g_{qj}x_{tj}-{\hat{c}}_{tqj}\log(g_{qj}x_{tj}\right))+\alpha \sum_{i}\sum_{t}^{T}\sum_{j}^{N}\frac{1}{2}\|{𝓛}_{i}{}_{\left(t\right)}^{\left(k+1\right)}-\Gamma_{itj}x_{tj}{\|}_{F}^{2}\;\;\;\;\;\;\;\;\;\;\;\;\;\;\;\;+\beta \sum_{t}^{T}\sum_{j}^{N}\frac{\eta }{2}{\left(D_{j}x_{tj}-\omega_{tj}{}^{\left(k+1\right)}\right)}^{2}+\nu_{tj}{}^{\left(k+1\right)}\left(\omega_{tj}{}^{\left(k+1\right)}-D_{j}x_{tj}\right)\;s.t.\;{\hat{c}}_{tqj}=\frac{g_{qj}x_{tj}{}^{\left(k\right)}}{\sum_{j}^{N}g_{qj}x_{tj}{}^{\left(k\right)}+r_{tq}+s_{tq}}y_{tq}.$$

Here, ${𝓛}_{i\left(t\right)}$ (or ${𝓛}_{i}\left(:,:,t\right)$) denotes the *t*-th frontal slice of tensor ${𝓛}_{i}$; $\Gamma_{itj}$ denotes the contribution weight for pixel $x_{tj}$ to tensor ${𝓧}_{i}$; $\omega_{tj}$ denotes the *j*-th element of $\omega_{t}$, and $D_{j}$ denotes the j-th column of the differential operator D; and $\nu_{tj}$ is the *j*-th element of multiplier $v_{t}$.

According to the M-step in Equation (18), we can harvest a unitary quadratic equation:

(25) $$A_{tj}{\left(x_{j}\right)}^{2}+B_{tj}x_{j}+C_{tj}=0\;s.t.{\;A}_{tj}=\alpha \sum_{i}\sum_{t}^{T}\Gamma_{itj}^{T}\Gamma_{itj}+\beta \eta \sum_{t}^{T}D_{j}^{T}D_{j}\;,\;\;\;\;\;\;\;\;\;\;\;C_{tj}=-\sum_{t}^{T}\sum_{q}^{M}{\hat{c}}_{tqj}\;{\;B}_{tj}=\sum_{t}^{T}\sum_{q}^{M}g_{qj}-\alpha \sum_{i}\sum_{t}^{T}\Gamma_{itj}^{T}{𝓛}_{i}{}_{\left(t\right)}^{\left(k+1\right)}\;-\sum_{t}^{T}\beta \eta D_{j}^{T}\omega_{tj}{}^{\left(k+1\right)}-D_{j}^{T}\nu_{tj}{}^{\left(k+1\right)}\;.$$

Therefore, $x_{tj}{}^{\left(k+1\right)}$ can be readily solved as the positive root of (25):

(26) $$x_{tj}^{\left(k+1\right)}=\frac{\left(-B_{tj}+\sqrt{B_{tj}{}^{2}-4A_{tj}C_{tj}}\right)}{2A_{tj}}.$$

In this X sub-problem, given that we do not have a close-formed solution for the PET reconstruction model [5], the X-subproblem is not fully solved by the employment of EM. However, due to the implementation of ADMM based optimization, the overall framework will converge, even if its sub-problems are not carried out exactly [30]. 

Algorithm 1 demonstrates the overall procedure of our proposed algorithm. It is noteworthy that, in the initialization step, we employed the filtered backward projection (FBP) [3] method to produce a ‘warm start’. By doing so, as we will show in the next section, iterative performance is notably improved.

## 4. Experiments and Results

In this section, we perform various experiments, in order to validate the qualitative and quantitative measures of the proposed method. Data on diversified photon counts, sizes, tracers, and structures are recovered by our proposed method and compared with the results of representative and state-of-the-art algorithms.

### 4.1. Implementations

#### 4.1.1. Evaluation Criteria

For the qualitative evaluation, randomly selected reconstructions are shown in this part, where the structural detail, noise level, and so on will be intuitively illustrated. For the quantitative evaluation, other than the *peak signal-to-noise ratio* (PSNR)), we also employ the relative bias and variance [45] as the indicators of the resolution and smoothness, respectively:

(27) $$\mathrm{Bias}=\left(\frac{1}{N_{t}}\right)\sum_{j}^{N_{t}}\frac{\left|x_{j}-{\hat{x}}_{j}\right|}{{\hat{x}}_{j}},$$

(28) $$\mathrm{Variance}=\left(\frac{1}{\left(N_{t}-1\right)}\right)\sum_{j}^{N_{t}}{\left(\frac{\left|x_{j}-\bar{x}\right|}{x_{j}}\right)}^{2},$$

where ${\hat{x}}_{j}$ denotes the ground truth in the *j*-th pixel; $\bar{x}$ denotes the mean value of the ROI; and $N_{t}$ denotes the total number of pixels in the given ROI. Unlike the PSNR, the smaller the relative bias and variance are, the better the reconstruction is. 

We also conduct the multiple simulations experiment. In this study, we employ the contrast recovery coefficient (CRC) and the standard deviation (STD) [16,46]:

(29) $$\mathrm{CRC}=\frac{1}{R}\sum_{r=1}^{R}\frac{\left|{\bar{S}}_{r}-{\bar{B}}_{r}\right|}{{\bar{B}}_{r}},$$

(30) $$\mathrm{STD}=\frac{1}{N_{B}}\sum_{j=1}^{N_{B}}\frac{\sqrt{\frac{1}{R-1}\sum_{r=1}^{R}\left(B_{r,j}-{\bar{B}}_{j}\right)}}{{\bar{B}}_{j}}.$$

Here, R=50 represents the number of realizations in the simulation. In Equation (29), ${\bar{S}}_{r}$ represents the mean value of the ROI in *r*-th realization and ${\bar{B}}_{r}$ represents the mean value of the background region in r-th realization. In Equation (30), $N_{B}$ denotes the total number of pixels in the background region; ${\bar{B}}_{j}=\left(1/R\right)\sum_{r=1}^{R}B_{r,j}$ denotes the mean value of *j*-th pixel in background region across *R* realizations.

For the real data, we adopt the *contrast to noise ratio* (CNR) [47]:

(31) $$CNR=\left(m_{ROI}-m_{background}\right)/SD_{background},$$

where the $m_{ROI}$ and $m_{background}$ represent the intensity of the ROI and background region respectively, and $SD_{background}$ is the standard deviation of the background region.

#### 4.1.2. Dataset

**Figure 4:**
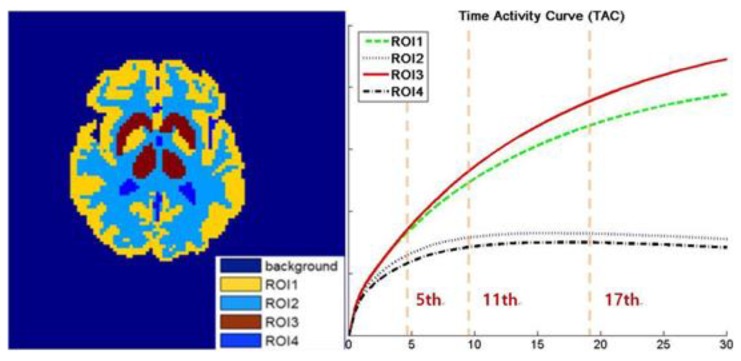
The ground truth of brain phantom. Left: the region of interest (ROI) map. Right: the time activity curves (TAC) of this image sequence, with the 5th, 11th, and 17th frames are labeled in the figure.

As shown in Figure 4, we mainly adopt a 64 × 64 sized Zubal brain phantom as the template and employ the 11C-dihydrotetrabenazine (denoted as DTBZ) as the tracer. The scanning procedure in simulated into 18 frames for a duration of 20 min with the corresponding TAC presented in Figure 1. Moreover, to validate the performance of the algorithms under a diversified tracer dose, the data are generated in 3 × 10^6^, 10^7^ and 3 × 10^7^ total photon counts over 18 frames. Furthermore, in the multiple realizations experiment, we generate total 100 realizations for 3 × 10^6^ and 3 × 10^7^ counted data (*R* = 50 for each simulation). In addition, all the simulated settings correspond to real cases.

Furthermore, 111 × 111 sized Zubal head phantoms [16] are recovered to validate the effectiveness of the proposed method on different tracers (18F-FDG) and image sizes. Moreover, real cardiac data are tested in this section. The data are scanned over 60 min by a Hamamatsu SHR-22000 (Hamamatsu Photonics K.K., Hamamatsu City, Japan). There are, overall, 19 frames, and each are scanned by 130 detector pairs from 192 angles.

#### 4.1.3. Comparative Algorithms

To evaluate the performance of the proposed algorithm, we introduce five representative algorithms in comparison (the maximum likelihood-expectation maximization (ML–EM) algorithm [5], the penalized weighted least square (PWLS) method [8], the total variation optimized by augmented Lagrangian (TV-AL) method [48], the penalized likelihood incremental optimization method regularized by hyperbolic potential function [45,49] (denoted as PLH-IO), and the spatial-temporal total variation (ST-TV) method [50]) proposed for dynamic PET reconstruction. 

#### 4.1.4. Parameters Setting

**Figure 5:**
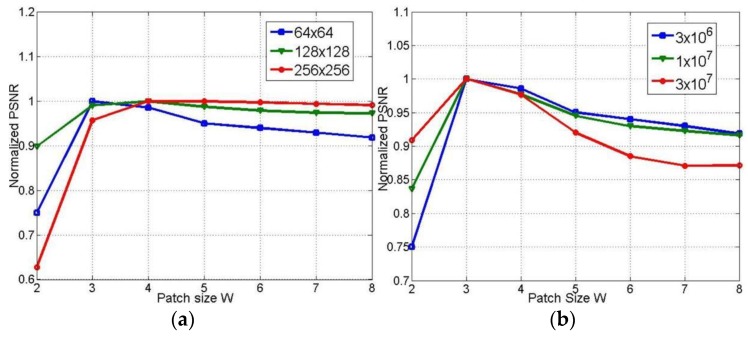
Normalized peak signal-to-noise ratio (PSNR) of reconstructions under different patch size W. (**a**) for images in different size, (**b**) for data under different photon count.

After deliberate examination, we set the weighting parameters as follows: the nonlocal tensor weight parameter α=1.7; TV weight parameter β=0.9; tensor thresholding parameter λ=2.5; and and parameter η=50. Another critical issue is the tensors’ sizes. As shown in Figure 5, the optimal patch size is 3 × 3. Thus, if the number of feature patches is set to 10 in the 18-frame sequence, each selected tensor’s size is 9 × 10 × 18. We set the maximum iteration to 500 for all methods in comparison.

#### 4.1.5. Experiment Description

**Figure 6:**
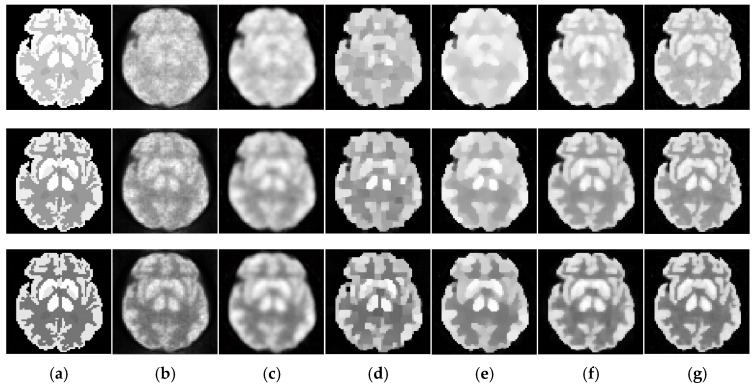
Dynamic brain phantom reconstructed by different algorithms. The total photon counts are $3\times 10^{7}$ over 18 image frames. From the first to the last row: the 5th, 11th and 17th frame. (**a**) ground truth, (**b**) ML-EM (16.01 dB, 16.41 dB, 15.92 dB), (**c**) PWLS (17.71dB, 16.88dB, 16.23dB), (**d**) TV-AL (18.49 dB, 18.76 dB, 18.52 dB), (**e**) PLH-IO (21.26 dB, 19.56 dB, 18.39 dB), (**f**) ST-TV (21.27 dB, 20.59 dB, 19.02 dB), (**g**) Ours (21.79 dB, 21.71 dB, 20.63 dB).

**Figure 7:**
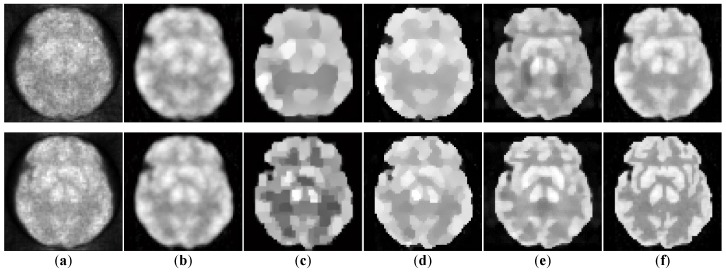
The simulated low-dosed images reconstructed by different algorithms. First row: the 11th frame under 186,337 photon counts. Second row: the 11th frame under 619,848 photon counts. (**a**) ML-EM (12.51 dB, 15.06 dB), (**b**) PWLS (15.89 dB, 16.67 dB), (**c**) TV-AL (16.09 dB, 16.45 dB), (**d**) PLH-IO (16.83 dB, 18.84 dB), (**e**) ST-TV (17.43 dB, 19.21 dB), (**f**) Ours (17.78 dB, 19.98 dB).

**Figure 8:**
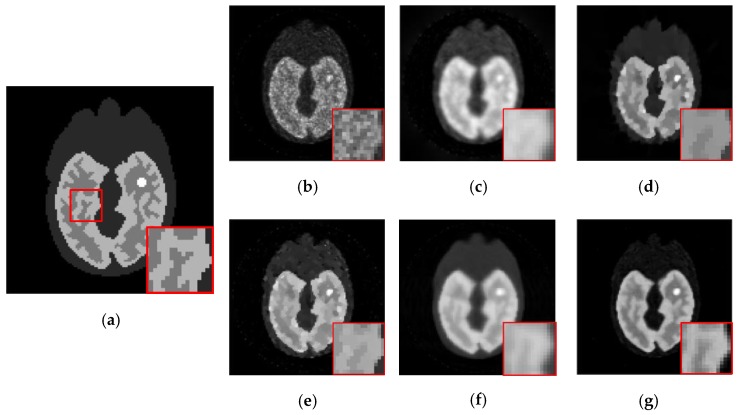
The 15th frame of 111 × 111 sized Zubal head phantom reconstructed by different algorithms. (**a**) ground truth, (**b**) ML-EM (18.19 dB), (**c**) PWLS (18.35 dB), (**d**)TV-AL (19.22 dB), (**e**) PLH-IO (19.17 dB), (**f**) ST-TV (19.45 dB), (**g**) Ours (19.73 dB).

**Figure 9:**
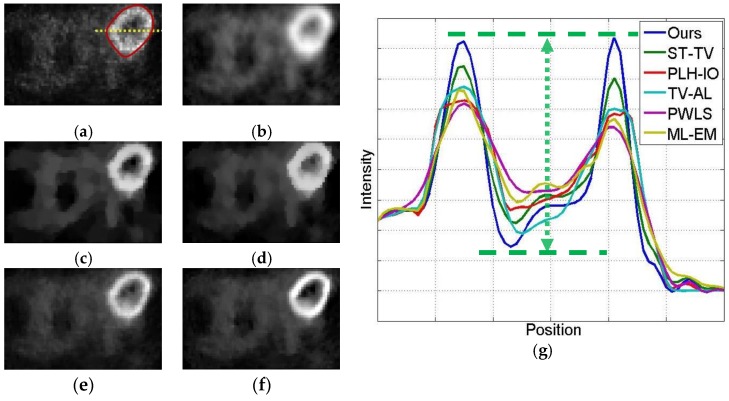
The 2nd frame of the dynamic cardiac real data, under 221,858 photon counts. The ROI is marked by a red circle. (**a**) ML-EM (CNR = 10.97), (**b**) PWLS (CNR = 13.72), (**c**) TV-AL(CNR = 18.46), (**d**) PLH-IO (CNR = 19.59), (**e**) ST-TV (CNR = 22.06), (**f**) Ours (CNR = 22.70), (**g**) the intensity profile across the ROI (the yellow line in the first graph). Our proposed method presents a superior contrast between the ROI and background region.

We evaluate our method both qualitatively and quantitatively on simulation and real PET data. We firstly compare the reconstructions for 64 × 64 sized Zubal brain data under 3 × 10^7^ total photon counts, and demonstrate 5th, 11th, and 17th frame in Figure 6. In Figure 7, we further compare the 11th reconstructed frames under lower-counted sequences: 3 × 10^6^ and 1 × 10^7^ photon counts. In addition, we recover the 111 × 111 sized Zubal head phantom to validate the performance under different sizes and TACs, as shown in Figure 8. For the real patient study, we demonstrate the first frame of dynamic cardiac PET reconstructions in Figure 9, and compute the CNR for each method.

**Figure 10:**
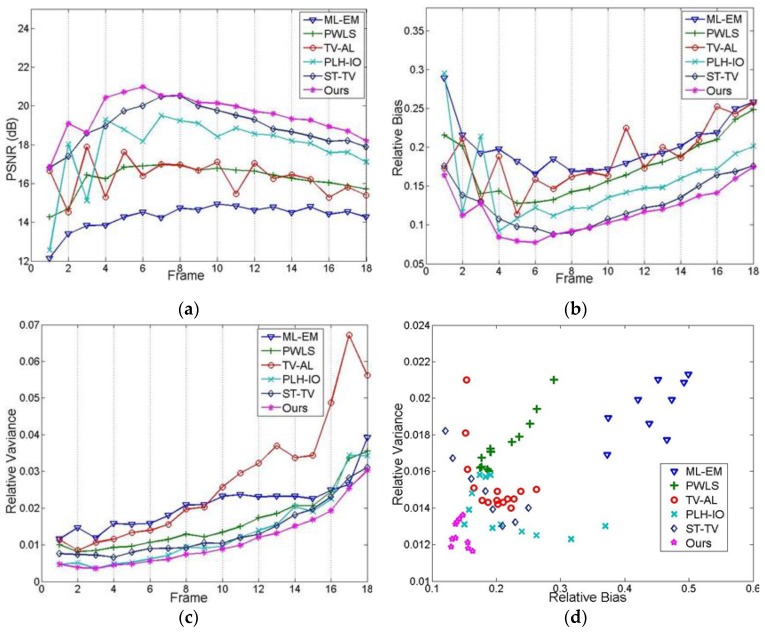
The quantitative evaluation for each image frame. (**a**) PSNR, (**b**) relative bias, (**c**) relative variance, (**d**) the experiment on the trade-off between image resolution and denoising performance.

In the quantitative evaluations, we firstly present the PSNR, relative bias and variance for data under 3 × 10^7^, 3 × 10^6^ and 1 × 10^7^ photon counts in Table 1; furthermore, we compute the relative bias and variance in each ROI for 3 × 10^6^ counted data and demonstrate them in Table 2. In Figure 10a–c, we compute the PSNR, relative bias, and variance for each frame in 1 × 10^7^ counted data. In Figure 10d, we demonstrate the performance of the trade-off between resolution and smoothness, by altering the parameters in each algorithm and plotting the bias and variance in each reconstruction. 

**Figure 11:**
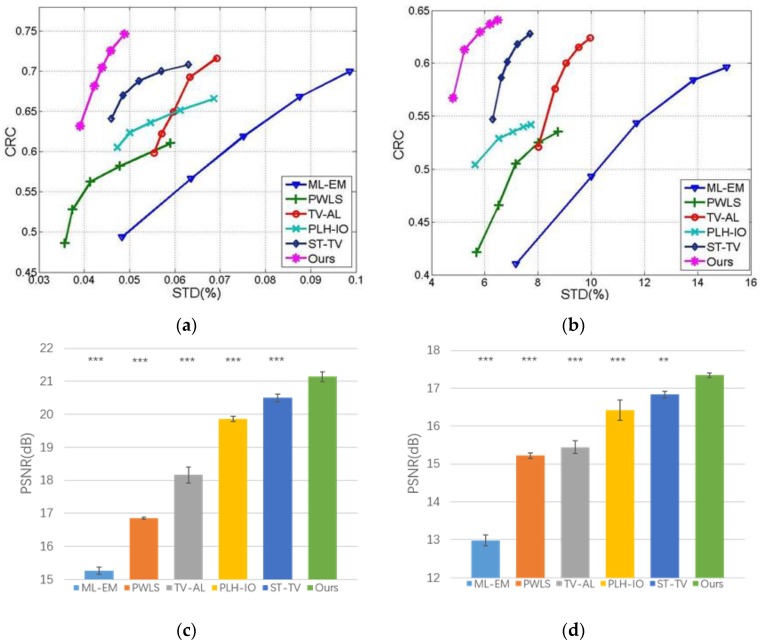
The statistical test on multiple realizations. First row: the CRC-STD curves for high-count and low-count simulations, with realization number R = 50 in each dataset. Second row: the PSNR bar plot and Wilcoxon rank sum test on multiple realizations datasets. Here ** and *** represent *p*-value < 0.01 and *p*-value < 0.001 respectively. (**a**) CRC-STD curves on 3 × 10^7^ dataset, (**b**) CRC-STD curves on the 3 × 10^6^ dataset, (**c**) Wilcoxon rank sum test on 3 × 10^7^ dataset, and (**d**) Wilcoxon rank sum test on the 3 × 10^6^ dataset.

Moreover, we conduct the experiment on multiple simulations. In our experiment, we run 100 realizations for 3 × 10^7^ and 3 × 10^6^ counted data, and draw the CRC-STD curves, as shown in Figure 11a–b. Accordingly, the CRC is computed from ROI 2, and the background STD is computed from ROI 4, which represents the white matter. In addition, we run the Wilcoxon rank sum test on the multiple realization test, as demonstrated in Figure 11c–d. In addition, we further examine the universality of the proposed method by reconstructing data under wider range of photon counts, as demonstrated in Figure 11a, from 1 × 10^6^ to 1 × 10^8^. Further discussions on convergence are illustrated in Figure 11b and the discussion section. 

### 4.2. Results

#### 4.2.1. Qualitative Evaluation

The initial experiment focuses on the resolution and the denoising performance throughout the temporal dimension. According to the TAC in Figure 4, the distinction of activity between different ROIs is inconspicuous in the early imaging stage, which consequently hampers the recovery of the structural information in the corresponding image frames. As demonstrated in Figure 6, the reconstructions of ML-EM and PWLS suffer from severe iterative noise and fail to recover a clear boundary between regions. On the other hand, the TV-AL and PLH-IO show more acceptable results for the 17th frame. However, when it comes to former frames, neither of these two methods are able to recover clear structures. Moreover, the TV-AL suffers from the staircase effect and artifacts, and PLH-IO tends to over-smooth the image. Although ST-TV improves the resolution by incorporating temporal information, it is still limited by recovering more detailed structures. In contrast, our proposed method manages to recover more detailed structures and less noise in reconstructing the brain phantom sequence under $3\times 10^{7}$ photon counts. This contrast is more distinctive in simulated low-dose images. As we can see in Figure 7, when recovering early frames in the low-count data, our proposed method is able to recover substantially clearer structures than those of other methods under similar noise levels. 

Meanwhile, our method also shows its universality in recovering sequences under different sizes and TACs. In this experiment, 111 × 111 sized Zubal head phantom data were tested in an 18F-FDG environment. In Figure 8, the 15th frame is randomly selected out of 24 frames. In addition, real patient data are tested. In Figure 9, the second sequence is shown, and the photon counts are around 2.2 × 10^5^. Obviously, our proposed method yields clearer boundaries and more conspicuous contrast between ROIs and the background. 

#### 4.2.2. Quantitative Evaluation

The quantitative measurements are also meticulously implemented. Table 1 demonstrates the average statistical values for the dynamic image sequences under diversified photon counts. According to the table, the proposed method enjoys a higher PSNR and lower relative bias and variance, revealing solid and substantial merits in structural enhancement, resolution improvement, and image denoising. Table 2 provides more detailed measurements for each ROI reconstruction. As shown in the table, the proposed method reconstructs images at a lower bias and variance in each ROI to vary the count level, which demonstrates better resolution and smoothness than those of comparable methods.

Other than spatial information, temporal trends are also considered in Figure 10, where Figure 10a–c presents the PSNR, bias, and variance for each frame. It can be easily observed that the proposed method, overall, has better results for each frame, which will facilitate the exploitation of temporal information. In addition, given the fact that regular reconstruction methods are largely based on the trade-off between the resolution and the noise level, we also implemented an experiment of this trade-off, correspondingly represented by the relative bias and the relative variance in Figure 10d. As we can see, apart from the relatively poorer performances of ML-EM and PWLS, both TV-AL and PLH-IO show a negative correlation between these two indexes. In contrast, the marks of our proposed method are densely concentrated in the bottom-left of this graph, which shows a better image quality and better compromise for the mentioned trade-off.

To better validate the proposed method, we further implement the experiments on multiple realizations. As demonstrated in Figure 11a–b, we run the experiments under high-count and low-count scenarios (3 × 10^7^ and 3 × 10^6^ counted data) and draw the correspondent CRC-STD curves, which illustrate the performance of compared methods. In these curves, each point corresponds to a certain setting for parameters in the relative method. According to the figures, the proposed method manages to recover higher CRC while keeping the background STD in a low level, which validates the stability of our method in reducing the noise while keeping the contrast distinctive. Moreover, we analyze the statistical performance of the Wilcoxon rank sum test on the multiple realizations data. As shown in Figure 11c–d, we compute the *p*-value between the proposed method and compared methods, in terms of PSNR on multi-simulation. In this study, the proposed method significantly outperforms the ST-TV in 3 × 10^6^ dataset, at *p*-value < 0.01; more distinctively, the proposed method outperforms other methods in 3 × 10^6^ dataset and all methods in 3 × 10^7^ dataset, at a *p*-value < 0.001.

#### 4.2.3. Robustness and Convergence Analysis

**Figure 12:**
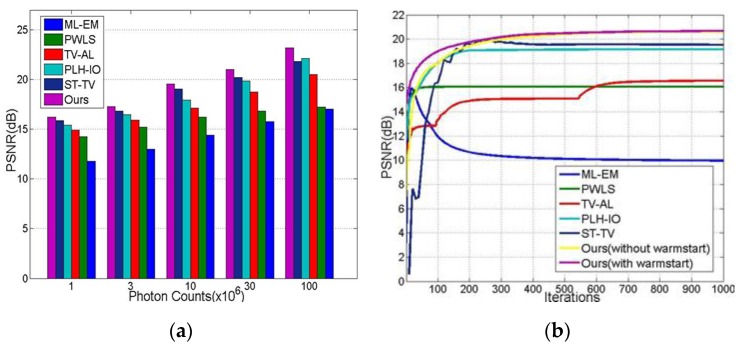
The robustness and convergence experiments. (**a**) the PSNR of reconstructed sequences under diversified photon counts; (**b**) the PSNR convergent trends along iterations.

The robustness and convergence experiments are demonstrated in this section. As we can see from Figure 12a, the proposed method presents better performance and robustness than other methods under a broad range of photon counts. 

For the convergence, Figure 12b demonstrates the PSNR for iterations of all tested methods. Specifically, we individually test our methods with and without a warmstart, which is mentioned in Section 3. Our warmstart-equipped method surpasses other methods in its convergence performance.

## 5. Discussion

Other than merely denoising, the proposed method simultaneously provides enhancement and completion of structural sparsity by introducing a 3D tensor based nonlocal low-rank constraint. Unlike the tracer kinetics based dynamic reconstruction method, the proposed method spontaneously exploits the inner temporal correlation within the sequence without the need for tracer information and model fitting. In fact, our proposed method not only managed to suppress noise while recovering at a high resolution, it also enhanced, and even completed, the structural information in the reconstruction sequence. 

This result is attributed to the tensor based low rank approximation. As presented in Figure 3, the third dimension of the ${𝓧}_{i}\in {\mathbb{R}}^{n\times m\times T}$ exists alongside the temporal information. By implementing the tensor based low rank constraint on each ${𝓧}_{i}$, the spatial information is spontaneously infiltrated from high-counted frames to low-counted frames, while keeping the voxels’ relative intensities and edge arrangements fixed. Considering this feature, a dynamic PET sequence is considered ideal for this framework, given the unchanged boundary and structures along the dynamic sequences. On the other hand, since noise is randomly and sparsely arranged in the PET sequence, it is ruled out as the sparse component in the low rank approximation. 

**Figure 13:**
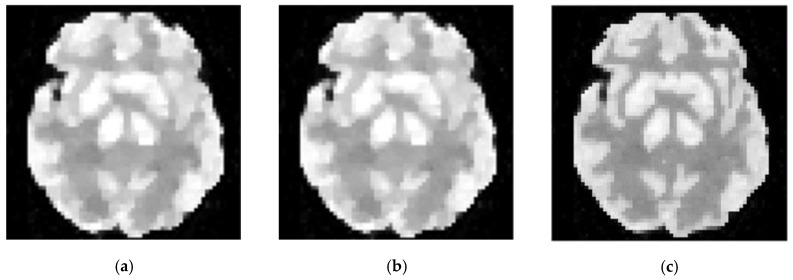
The comparison of reconstructed images under different settings of hyper-parameters. (**a**) reconstruction constrained by TV regularization (by setting α = 0), (**b**) reconstruction constrained by nonlocal low rank tensor (by setting β = 0), (**c**) image reconstructed by proposed methods (α = 2.5, β = 1.4).

Furthermore, we demonstrate the contribution of different regularization components in Figure 13 by setting various hyper-parameters. According to the figure, tensor constraints can successfully recover detailed structures but are slightly limited in smoothing an image under low counts. Fortunately, the employment of a TV constraint compensates for this issue, as demonstrated in Figure 13c.

Nevertheless, there are still several concerns in this study. Firstly, for the data in unwilling but conspicuous motion, the proposed method is limited in harvesting temporal correlation, though other dynamic PET algorithms also suffer from this issue, to the best of our knowledge. Currently, the optimal solution is to conduct motion correction before reconstruction. Secondly, since 3D structural nonlocal features are not well proven in the computer vision community, this method is not guaranteed to function well in 3D PET reconstruction. However, we still provide two solutions for applying the proposed method in 3D data at the current stage: (1) Conduct the method slice by slice; and (2) rebin the data into a 2D form before reconstruction. 

The last issue focuses on the computational cost. For simulated data in Figure 4, the computational time for each method is demonstrated in Table 3. Here, the computational experiments are implemented under Matlab R2014a (Mathworks, Natick, MA., USA), on the same desktop with an Intel Core i7-4720HQ CPU (Santa Clara, CA, USA) @2.60 GHz and 8 GB RAM. We have to concede that, compared with the traditional pixel-based algorithms, the computational cost is inevitable in the proposed method, due to the multiple decompositions for feature tensors generated by feature cubes (or patches in 2D situation [23,24]). To address this issue, we will continue optimizing the proposed algorithm and employing other algorithms, such as TCTF [51].

In addition, the choice for the tensor decomposition model is still open in our future work. The T-SVD based method is proved effective in our work and [34,35,36], yet, strictly speaking, its tubal based rank is the analogous rank extended from SVD. In our future work, we will further analyze the data-structures, explore the feasibilities of other potential models, i.e., CP and Tucker rank [31], and testify the applicabilities of the latest proposed CP rank based methods [52,53] as well as Tucker rank based methods [54,55]).

## 6. Conclusions

In this paper, we provide a novel tensor based nonlocal low-rank framework for dynamic PET reconstruction. By introducing a nonlocal featured tensor and applying the t-SVT in low-rank tensor approximation, the image structures are efficiently enhanced while effectively depressing the noise. More significantly, structural information is further completed by other frames in an interactive way, thereby compromising the conflict between spatial and temporal resolution. On the other hand, accompanied by the TV term denoising (from a local and pixel-based perspective), the regularizations are firstly integrated in the Poisson reconstruction model and efficaciously optimized in a distributed framework.

## Figures and Tables

**Table 1:** Average value of statistical evaluation terms for reconstructed Brain Phantom sequences under different photon counts.

Algorithm	PSNR (dB)			Relative Bias			Relative Variance		
	3 × 10^6^	1 × 10^7^	3 × 10^7^	3 × 10^6^	1 × 10^7^	3 × 10^7^	3 × 10^6^	1 × 10^7^	3 × 10^7^
ML-EM	13.00	14.39	15.75	0.2374	0.2024	0.1742	0.0252	0.0147	0.0138
PWLS	15.20	16.25	16.81	0.2054	0.1747	0.1627	0.0217	0.0162	0.0154
TV-AL	15.91	17.13	18.76	0.1804	0.1548	0.1286	0.0303	0.0161	0.0152
PLH-IO	16.48	17.92	19.87	0.1883	0.1539	0.1225	0.0205	0.0131	0.0115
ST-TV	16.85	19.07	20.21	0.1725	0.1359	0.1139	0.0161	0.0125	0.0097
**Ours**	**17.29**	**19.54**	**21.03**	**0.1609**	**0.1172**	**0.0983**	**0.0148**	**0.0111**	**0.0083**

**Table 2:** Average value of relative bias and variance in each region of interest (ROI). 3 × 10^6^ photon counted data are tested and shown.

Algorithm	Relative Bias					Relative Variance				
	Whole	ROI1	ROI2	ROI3	ROI4	Whole	ROI1	ROI2	ROI3	ROI4
ML-EM	0.2374	0.2353	0.3909	0.4407	0.2654	0.0252	0.0260	0.0105	0.0292	0.0410
PWLS	0.2054	0.1955	0.1853	0.2381	0.1801	0.0217	0.0377	0.0054	0.0176	0.0217
TV-AL	0.1804	0.1905	0.1758	0.2114	0.1522	0.0303	0.0464	0.0056	0.0215	0.0303
PLH-IO	0.1883	0.2601	0.1560	0.1680	0.2077	0.0205	0.0285	0.0035	0.0205	0.0205
ST-TV	0.1725	0.2001	0.1388	0.1700	0.1791	0.0161	0.0210	0.0042	0.0151	0.0190
**Ours**	**0.1609**	**0.1864**	**0.1235**	**0.1591**	**0.1671**	**0.0148**	**0.0203**	**0.0035**	**0.0118**	**0.0186**

**Table 3:** Computational time for each method.

Method	ML-EM	PWLS	TV-AL	PLH-IO	ST-TV	Ours
Computational time (s/iteration)	0.04375	0.3163	0.07935	0.3507	0.1639	4.379
